# Powering HEARTS 2.0: how digital health and AI could transform integrated cardio-kidney-metabolic care in the Americas

**DOI:** 10.1016/j.lana.2026.101645

**Published:** 2026-09-22

**Authors:** Sandeep P. Kishore, Pranay Narang, Ida Sim

**Affiliations:** a Department of Medicine, University of California, San Francisco, San Francisco, CA, USA; b University of California, Berkeley/UCSF Joint Medical Program, Berkeley, CA, USA; c Philip R. Lee Institute for Health Policy Studies, University of California, San Francisco, San Francisco, CA, USA; d Computational Precision Health, University of California, San Francisco, San Francisco, CA, USA

Ordunez and Barbosa da Silva Jr. clearly state that the obstacle to scaling integrated cardiovascular–kidney–metabolic (CKM) care in the Americas is implementation.^1^ Cardiovascular disease causes over two million deaths annually in the region, roughly a quarter of the total,^2^^,^^3^ yet hypertension control in Latin America and the Caribbean averages just 28.7%.^4^ Their six-lever framework is a concrete approach to closing that gap. We wish to press on the lever they themselves identify as a critical constraint, and which the current moment makes the most consequential: digital health infrastructure, now amplified by artificial intelligence (AI).

The authors are candid that underdeveloped, fragmented information systems are a key bottleneck to scaling HEARTS, that interoperable electronic records and patient portals remain scarce, and that poorly integrated tools can add administrative burden rather than relieve it. Digital infrastructure should be understood, as the authors put it, as a critical component of improving performance, for clinicians and patients alike. In the era of AI, it can move from merely supporting the transformation to powering it.

Two technical advances can help. First, digital health platforms have matured to provide interoperable infrastructure capable of integrating multimodal clinical and wearable data. For instance, JupyterHealth–an open-source, vendor-agnostic platform with which SPK and IS are founders–integrates real-time health data from wearables and electronic health records (EHRs) into unified, AI-enabled environment. Already oriented toward metabolic disease, it supports the integration of data streams central to CKM care, including validated blood-pressure sensors and continuous glucose monitors, into EHRs.^5^ By providing privacy-preserving, shared, reproducible, and vendor-neutral infrastructure, the technology enables EHRs, devices, and software applications to interoperate within a common ecosystem, which helps reduce the fragmentation challenges highlighted by the authors. Second, the incorporation of AI into open platforms enables underlying data inputs, workflows, and model outputs to be exposed and assessed. This ultimately enables health systems and clinicians to validate and continuously monitor risk of the CKM care cascade while evaluating model performance, bias, and equity. In doing so, technology can help strengthen the governance and clinical oversight the authors rightly advocate for.^6^

A key test of technology's potential is its reach into vulnerable settings including rural and low-income populations that health systems have long missed. In such settings, AI-powered platforms can be used in several ways. AI-enabled, pathway-driven decision support embeds evidence-based CKM guidelines and allows nurses, pharmacists, and community health workers to deliver guideline-concordant CKM care on scale. New remote monitoring technologies make longitudinal measurements more accessible by bringing blood pressure, glucose, and other physiologic monitoring into patients' homes, reducing dependence on in-person visits and expanding access for populations who cannot easily travel. Multilingual, low-literacy, and voice-enabled interfaces, including in Indigenous languages, make self-management more accessible across diverse populations. At the health system level, population dashboards and targeted outreach can surface individuals missed and prioritize scarce specialist capacity for the highest-risk patients. The alternative is that the same tools can widen inequity if built on closed systems or trained on unrepresentative data.^7^

Digital transformation helps enable other goals for CKM. For instance, standardized clinical pathways are the operational backbone of HEARTS 2.0, translating dispersed CKM guidelines into simplified, protocol-driven care. Here, digital functions can help measure fidelity and outcomes. Forecasting and predictable demand signals, generated by standardized pathways and digital records, are what turn that potential into reliable access. The technological foundations (e.g. open, interoperable digital health platforms; multimodal data integration and AI-enabled tools that broaden access to care) now exist to enable HEARTS 2.0 to scale efficiently through new care models of integrated CKM care.

## Contributors

SPK prepared the first draft and PNS and IS have edited this manuscript. All authors revised and approved the final version of the manuscript.

## Declaration of interests

SPK reported receiving consulting fees from Redesign Health; grants from CareX.AI and the Laude Institute; and having equity in CareX.AI. PN declared no conflicts of interest. IS reports consulting fees from Vivli, Inc and Apple, Inc; grants from Laude Institute; Stock in CareX.AI and Knit Health, Inc. other financial or non-financial interests in JupyterHealth.
